# Ophthalmological and epidemiological profiles of Brazilian patients with philadelphia-negative Myeloproliferative neoplasms

**DOI:** 10.1016/j.htct.2026.106536

**Published:** 2026-09-18

**Authors:** Rômulo Piloni-Parreira, Melaine Stefane Barbosa, Laís Lauria Neves, Hernani Lopes Santana, João Marcos Ranyere da Silva Rodrigues, Renato Sampaio Tavares, David Leonardo Cruvinel Isaac, Marcos Pereira de Ávila

**Affiliations:** a Centro de Referência em Oftalmologia, Federal University of Goiás, Goiânia, Goiás, Brazil; b Department of Hematology, Federal University of Goiás, Goiânia, Goiás, Brazil

**Keywords:** Myeloproliferative disorders, Polycythemia vera, Primary myelofibrosis, Polycythemia, Retinal hemorrhage, Age-related macular degeneration

## Abstract

**Introduction:**

Philadelphia-negative myeloproliferative neoplasms comprise a rare and biologically heterogeneous spectrum of hematopoietic stem cell malignancies. According to established clinicopathological criteria, these entities are principally categorized as polycythemia vera, essential thrombocythemia, or primary myelofibrosis. Ophthalmic manifestations are common in hematological diseases, yet they remain under-researched in this patient population.

**Methods:**

The purpose of this study is to analyze the ophthalmological and epidemiological profile of patients, describing ocular findings of Philadelphia-negative Myeloproliferative Neoplasms. Twenty-one individuals (forty-two eyes) with a Philadelphia-negative myeloproliferative neoplasm had their medical records selected, analyzed and relevant data collected. They were subsequently subjected to a complete ophthalmological evaluation, with assessment of visual acuity, biomicroscopy and fundoscopy employing wide-field color retinography and wide-field fundus autofluorescence examinations.

**Results:**

The mean age of participants was approximately 60 years, with a white demographic predominance and no sex bias. Most patients achieved early clinical diagnosis and effective disease control, with a high frequency harboring the *JAK2* V617F mutation. Patients reported no subjective complaints of low visual acuity, and posterior segment involvement was sparse. Retinal hemorrhages were uncommon, occurring predominantly in polycythemia vera due to hyperviscosity or microvascular thrombosis in the absence of confounding ocular or systemic conditions. Fundoscopic lesions were mostly driven by comorbid diagnoses, specifically age-related macular degeneration (19.4%) and hypertensive retinopathy (9.5%).

**Conclusion:**

Brazilian patients with well-controlled Ph-NMPs and early diagnosis rarely exhibit primary retinal complications or subjective low visual acuity. Retinal manifestations, particularly hemorrhages, are infrequent despite high rates of the *JAK2* V617F mutation. Ocular findings in this population are more commonly driven by concurrent systemic or degenerative conditions, notably age-related macular degeneration.

## Introduction

Philadelphia-negative myeloproliferative neoplasms (Ph-MPNs) are rare hematological malignancies originating from somatic mutations in hematopoietic stem cells. The primary clinical complications include thrombosis, hemorrhage, and leukemic transformation. The three main subtypes are polycythemia vera (PV), essential thrombocythemia (ET), and primary myelofibrosis (PMF) [1,2]. Classification and diagnostic criteria have undergone significant changes in recent years, reflecting an increased understanding of clinical and pathological characteristics. Recent guidelines emphasize the importance of a multidisciplinary approach due to hematological abnormalities, microvascular disorders, and hyperviscosity, which can affect various organs, including the eyes, with complications that threaten vision potentially preceding extraocular complications [2].

Ocular manifestations in Ph-MPNs are rarely reported in the literature, with existing evidence restricted to case reports or broader studies covering heterogeneous hematological conditions. Despite this gap, ocular manifestations involving hematological diseases in general occur in 90% of cases, depending on the hematological disease [3]. As far as we know, there are few studies covering ophthalmological alterations associated with Ph-NMPs, despite their clinical relevance [2]. This scarcity of research is presumably attributable to the low prevalence of these conditions relative to other ocular diseases, as well as the perception that they rarely lead to severe visual impairment. Ophthalmological alterations related to hematological diseases are common events, even without initial ophthalmological symptoms, making early diagnosis uncommon [2,[3], [4], [5]], with the conjunctiva and retina being the sites of greatest involvement. Unexplained retinal changes may originate from underlying hematological diseases that should be excluded [2,3].

The pathophysiology of ocular manifestations in ET and PV involves thrombocytosis, erythrocytosis, leukocytosis, and hyperviscosity, which can lead to vascular occlusions and irreversible visual loss [2]. PMF may present with leukocytosis and thrombocytosis, accompanied by milder systemic symptoms. Ophthalmic features reported in PMF include retinopathy, ischemia, neovascularization, hemorrhages, and cotton-wool spots, whereas vascular occlusions remain rare [6], reflecting the heterogeneity across published studies.

This study aims to provide a clearer understanding of the ocular manifestations of Ph-MPNs to better guide ophthalmologists in clinical practice.

## Methods

This collaborative study between the departments of ophthalmology and hematology was conducted in accordance with the Declaration of Helsinki and approved by the institutional ethics committee. Medical records were evaluated to identify patients with diagnoses of hematological diseases and collect clinical and sociodemographic data. Subsequently, patients underwent ophthalmological examinations, including corrected visual acuity (CVA), biomicroscopy, tonometry, gonioscopy, wide-field color retinography (WFCR), and wide-field fundus autofluorescence (WFFA) (Figure 1). First, visual acuity was evaluated using the Early Treatment of Diabetic Retinopathy Study (ETDRS) chart with optical correction, measured via an autorefractor (Topcon KR-8800®, Tokyo, Japan) and a phoropter (Greens Topcon CV-5000®, Tokyo, Japan). This was followed by slit-lamp biomicroscopy (Zeiss SL-800®, Germany), applanation tonometry using a Goldmann tonometer (Zeiss Visuplan 500®, Germany), and gonioscopy with a four-mirror lens (Volk G-4 Mirror Gonio Lens®, Ohio, USA). Pharmacological mydriasis was achieved by instilling 1% tropicamide and 2.5% phenylephrine eye drops three times at 10-minute intervals. The images were analyzed by four retinal specialists, who evaluated vascular changes, retinal hemorrhages, and cotton-wool spots. All data were de-identified using numerical codes prior to collection and analysis.

**Figure 1:**
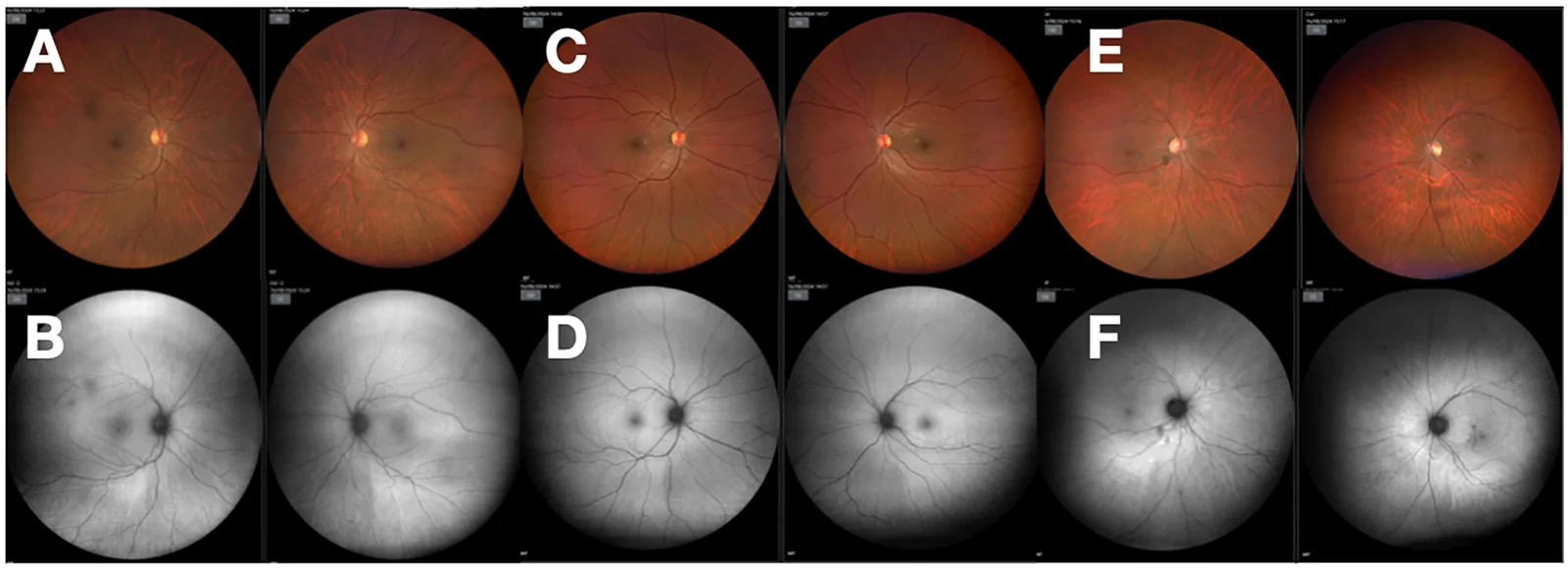
Wide-field color retinography (WFCR) and wide-field fundus autofluorescence (WFFA) examinations of patients with Philadelphia-negative myeloproliferative neoplasms. (A: WFCR of an individual with no abnormalities; B: WFFA of an individual with no abnormalities; C: WFCR of an individual with mild retinal hemorrhage; D: WFFA with mild retinal hemorrhage; E: WFCR of an individual with mild macular drusen; F: WFFA of an individual with mild macular drusen)

Patients aged 18 years or older with a confirmed diagnosis of Ph-MPN of at least six months' duration were included if they completed adequate ophthalmological examinations and provided written informed consent. Exclusion criteria comprised previous ocular surgery and uncontrolled systemic conditions that could confound study outcomes. Statistical analyses were performed using IBM SPSS software *(*version 26.0). Continuous variables were assessed for normality with normally distributed data being expressed as means and standard deviation, whereas categorical variables were presented as absolute and relative frequencies.

## Results

Of the 458 medical records of patients with hematological diseases analyzed, 81 (17.69%) had a Ph-MPN (PV, ET, or PMF). When contacted, nine (11.12%) had died, 19 (23.45%) did not respond, and 14 (17.28%) did not want to participate. Therefore, 39 (48.15%) patients agreed, however, two (5.13%) withdrew and 17 (41.02%) were excluded for not presenting adequate imaging exams, thus, the final sample consisted of 21 patients (42 eyes).

The mean cohort age was 62.1 ± 13.5 years, ranging from 32 to 80 years (both female). Most patients were older than 60 years (66.7%). Age stratification demonstrated that 28.57%, 14.29%, and 4.76% of patients were <50, <45, and <40 years of age, respectively. Within the subgroup aged under 50 years, PMF accounted for 66.7% of the cases and PV for 33.3%; similarly, PMF comprised 66.7% of patients under 45 years. A sociodemographic evaluation revealed a slight female predominance in the cohort (52.4%). When stratified by clinical diagnosis, gender distribution was as follows: ET, five (23.8%) patients (3 males, 2 females); PV, seven (33.3%) patients (2 males, 5 females); and PMF, nine (42.9%) patients (5 males, 4 females).

Regarding racial demographics, White patients predominated (66.7%), followed by mixed-race patients (28.6%). Crosstabulation by sex demonstrated that seven females (63.6%) were White and four (36.4%) were admixed/mixed-race, whereas male patients comprised seven Caucasian (70.0%), two admixed/mixed-race (20.0%), and one Black (10.0%) individual. Stratification by specific pathology revealed the following racial distribution: for ET, four patients (80.0%) were Caucasian and one (20.0%) was admixed/mixed-race; for PMF, six (66.7%) were Caucasian and three (33.3%) were admixed/mixed-race; for PV, four patients (57.1%) were Caucasian, two (28.6%) were mixed-race, and one (14.3%) was Black.

Regarding occupational background, no single occupation predominated; the most frequently reported were farmer (n = 3; 14.3%), and receptionist, general services worker, and domestic worker (n = 2, 9.5% each). The clinical characteristics of the participants (n = 21; 42 eyes) are summarized in Table 1.

**Table 1:** Clinical and demographic characteristics of patients with Philadelphia-negative myeloproliferative neoplasms.

Parameter	
**Age group** - n (%)	
32 to 59	14 (33.3)
60 or older	28 (66.7)
**Age** Years - Mean ± SD	62.1 ± 13.5
**Gender** - n (%)	
Female	22 (52.4)
Male	20 (47.6)
**Racial background** - n (%)	
White	28 (66.7)
Black	2 (4.8)
Mixed	12 (28.6)
**Time after diagnosis** - n (%)	
≤ 1 year	10 (23.8)
2-5 years	14 (33.3)
6-10 years	10 (23.8)
11-18 years	8 (19.0)
**Clinical treatment** - n (%)	
Disease control	32 (76.2)
No control	10 (23.8)
**Splenomegaly** - n (%)	
No	26 (61.9)
Yes	16 (38.1)

SD: standard deviation

At the time of diagnosis, the mean age of the cohort was 56.4 years, (range: 29-75 years both female). Age-stratified analysis revealed marked shifts compared to current age distribution: the proportion of individuals aged <50, <45, and <40 years at diagnosis rose to 38.1%, 28.6%, and 23.8%, respectively (compared with 28.6%, 14.3%, and 4.8% for current age). The majority of patients (76.2%) were receiving active treatment. Splenomegaly was observed in 38.1% of all cases, including all patients who were not currently undergoing treatment. Among patients younger than 40 years of age, 80.0% achieved clinical disease control and 60.0% presented without splenomegaly.

Regarding ophthalmological parameters, the mean best-corrected visual acuity (BCVA) was 0.15 ± 0.23 logMAR and the mean intraocular pressure (IOP) was 13.8 ± 3.1 mmHg (Table 2). All eyes presented with an open angle on gonioscopy. Slit-lamp biomicroscopy demonstrated a normal anterior segment in 47.6% of the eyes, while 21.4% were pseudophakic. Among anterior segment pathological findings, cataracts were most prevalent (21.4%), followed by posterior capsule opacification (4.8%) and pterygium (4.8%). Imaging using WFFA revealed normal retinal findings in 76.2% of eyes, whereas WFCR demonstrated absence of pathologies in 52.4%. Key structural abnormalities encompassed macular drusen (19.0%), vascular alterations (9.5%), and hard exudates and hemorrhages (2.4% each). Stratified by clinical diagnosis, age-related macular degeneration (AMD) was identified in 19.0% of eyes, hypertensive retinopathy in 9.5%, and cataract in 7.1%. Notably, AMD represented 47.1% of the total ocular disease burden in this Ph-MPN cohort.

**Table 2:** Characterization of the ophthalmological profile of patients with Philadelphia-negative myeloproliferative neoplasms.

Eye - n (%)	(n = 42)
Right	21 (50.0)
Left	21 (50.0)
**Ophthalmological Data** - Mean ± SD	
BCVA (logMAR)	0.15 ± 0.23
IOP (mmHg)	13.79 ± 3.14

SD: standard deviation; CVA: corrected visual acuity; BCVA: best corrected visual acuity; IOP: intraocular pressure.

Characterization of the ophthalmological profile of patients with Philadelphia-negative Myeloproliferative Neoplasms in terms of laterality, best corrected visual acuity, and intraocular pressure measured by tonometry

An assessment of comorbidity prevalence demonstrated systemic arterial hypertension in 42.9% of patients, followed by dyslipidemia (28.6%), hypothyroidism (28.6%), osteoporosis (19.0%), stroke (14.3%), and cardiovascular disease (14.3%); 14.3% of patients had no comorbid conditions (Table 3). Categorically, 83.3% of diagnosed conditions were associated with systemic inflammatory complications characteristic of Ph-MPN, whereas 16.7% represented isolated non-inflammatory comorbidities. Genetic testing for *JAK2* V617F was performed in 90.5% of patients, among whom 78.9% harbored the mutation and 21.1% lacked a detectable mutation. The *JAK2* V617F mutation was present in 75.0% of ET cases, 83.3% of PV cases, and 88.9% of PMF cases. Testing for the *CALR* gene was conducted in 47.6% of patients, revealing no mutation in 40.0%, a Type 1 mutation in 30.0%, a Type 2 mutation in 15.0%, and inconclusive results in 15.0%. *MPL* testing was performed in 14.3% of patients, identifying the p.W515K mutation.

**Table 3:** Characterization of the ophthalmological profile, detailing the findings of the examinations and ophthalmological diseases, in addition to the prevalence of comorbidities and the use of medication for Philadelphia-negative myeloproliferative neoplasms.

Indicator	n (%)	Indicator	n (%)
**Biomicroscopy**		**Comorbidity**	
Cataract	9 (21.4)	Stroke	6 (14.3)
Pseudophakia	9 (21.4)	Alzheimer	2 (4.8)
Posterior capsule opacification	2 (4.8)	Rheumatoid arthritis	2 (4.8)
Pterygium	2 (4.8)	Asthma	4 (9.5)
No changes	20 (47.6)	Heart disease	6 (14.3)
**Fundus autofluorescence (WFFA)**		Diabetes Mellitus	4 (9.5)
Hypoautofluorescent fovea	2 (4.8)	Chronic Kidney Disease	2 (4.8)
Macular hyperautofluorescence spots	4 (9.5)	Depression	4 (9.5)
Hyper and hypoautofluorescent spots	4 (9.5)	Dyslipidemia	12 (28.6)
No changes	32 (76.2)	Glaucoma	2 (4.8)
**Retinography (WFCR)**		Hypertension	18 (42.9)
Vascular changes	4 (9.5)	Liver disease	4 (9.5)
Macular drusen	4 (9.5)	Hypothyroidism	12 (28.6)
Increased excavation	2 (4.8)	Osteoporosis	8 (19.0)
Hard perimacular exudates	1 (2.4)	Psoriasis	2 (4.8)
Blotchy hemorrhagic spot	1 (2.4)	Heavy smoker and drinker	2 (4.8)
RPE rarefaction	2 (4.8)	No comorbidities	6 (14.3)
RPE rarefaction + macular drusen	2 (4.8)	**Medication**	
No changes	26 (61.9)	ASA	20 (47.6)
**Ophthalmological diseases**		Clopidogrel	2 (4.8)
Cataract	3 (7.1)	Hydroxyurea	24 (57.1)
AMD	2 (4.8)	Prednisone	2 (4.8)
AMD + Cataract	4 (9.5)	Ruxolitinib	8 (19.0)
Glaucoma	4 (9.5)	Does not use	2 (4.8)
Hypertensive retinopathy	2 (4.8)		
Hypertensive Retinopathy + Cataract	2 (4.8)		
No diagnosis	25 (59.5)		

AMD: age-related macular degeneration

An association was observed between WFCR abnormalities (n = 6) and both clinical management and time since diagnosis; however, neither association reached statistical significance (Table 4).

**Table 4:** Results of the association between changes in retinography (WFCR) with clinical findings and time of diagnosis.

Parameter	Wide-field Color Retinography – n (%)			p-value
	No	Yes	Total	
**Clinical treatment**				
Disease control	28 (77.8)	4 (66.7)	32 (76.2)	0.554
No control	8 (22.2)	2 (33.3)	10 (23.8)	
**Time after Diagnosis**				
≤1 year	10 (27.8)	0 (0.0)	10 (23.8)	0.266
2-5 years	12 (33.3)	2 (33.3)	14 (33.3)	
6-10 years	8 (22.2)	2 (33.3)	10 (23.8)	
11-18 years old	6 (16.7)	2 (33.3)	8 (19.0)	

## Discussion

The study population demonstrated a primary concentration of individuals aged over 60 years, whereas young-onset disease was uncommon (4.8% under 40 years; 14.3% under 45 years). These findings broadly align with large-scale international cohorts: two separate studies comprising 11,000 and 726 patients with MPNs documented <40 and <45 age proportions of 4.2% and 18.8%, respectively [7,8]. While a distinct report of 630 patients identified a higher subgroup under 40 years (27%) [9], the findings of this study reinforce overall demographic consistency within the Ph-MPN literature. Improved diagnostic infrastructure likely contributes to earlier identification in diverse cohorts. Despite the characteristically admixed background of the Brazilian population, clinical manifestations in this cohort align with findings from homogeneous reference populations, even though 66.7% of participants self-identified as White. Existing epidemiological data indicate that the incidence of MPN among Hispanic White individuals may be lower compared to their non-Hispanic White counterparts [10].

The mean overall age was 62.1 years, whereas the mean age at diagnosis was 56.4 years. When stratified by age brackets, the proportion of patients diagnosed under 45 years of age increased from 14.3% to 28.6%, and those under 40 years increased from 4.8% to 23.8%. These findings support the growing consensus that myeloproliferative neoplasms are increasingly identified in younger patients. Diagnosis in this demographic remains particularly challenging, as approximately half of young patients are asymptomatic at presentation [11]. Consequently, tailored management strategies are required to address potential treatment non-adherence [12], as well as the heightened long-term risks of clonal evolution and thrombotic events.

Access to comprehensive molecular diagnostics poses a persistent hurdle in Brazilian public healthcare. While most patients aged under 50 years received *JAK2* V617F screening, testing for the *CALR* and *MPL* genes was rarely performed, underscoring systemic diagnostic vulnerabilities. Notably, the majority of younger patients presented with controlled disease and an absence of splenomegaly, a clinical pattern characteristic of early or prefibrotic PMF [13].

Age stratification at diagnosis yielded mean ages of 66, 57, and 50 years for ET, PV, and PMF, respectively, whereas reference literature reports corresponding mean ages of 65, 67, and 69 years. The mean age at diagnosis in the present cohort aligns with established benchmarks for ET, but is lower by 10 years for PV and 19 years for PMF. This trend is likely attributable to earlier clinical detection, given that comparison cohorts reflect the 2001–2012 era when diagnostic criteria for ET were relatively static compared to the evolving definitions for PV and PMF [1,3,10,11,[14], [15], [16], [17]].

Genetic sequencing has improved our understanding of clinical and pathological characteristics [18], especially in respect to the JAK2 V617F mutation. Reported mutational frequencies vary, with the JAK2 mutation present in 95–100% of PV cases versus 50–60% of ET and PMF cases [8]. In this cohort, JAK2 V617F was detected in 60.0% of ET, 83.3% of PV, and 88.9% of PMF cases. While the ET mutation rate (60.0%) is consistent with established literature, the PV rate (83.3%) falls slightly below expected historical benchmarks, although this gap may be artificially widened by the lack of testing in a subset of patients. Results for PMF diverged from expected values, as 100% of these patients underwent *JAK2* testing and all but one tested positive for the *JAK2* V617F mutation. Identification of the *JAK2* V617F variant, one of the most frequent driver mutations [8,11], is clinically significant, as it correlates with adverse outcomes, including secondary hematologic transformation, hemorrhagic complications, and the chronic inflammatory state characteristic of Ph-MPNs [19]. In comparison with *JAK2* mutations, *CALR* variants are associated with a more favorable clinical trajectory, demonstrating a reduced incidence of thrombotic events in ET and prolonged survival outcomes in PMF [[20], [21]].

This patient cohort exhibited multiple concurrent systemic comorbidities, which could introduce confounding factors into the interpretation of ophthalmologic findings. However, identifying individuals with Ph-NMPs in the absence of comorbidities is practically unfeasible, as many of these conditions share key inflammatory signaling pathways. These comorbid states are intrinsically linked to the sustained systemic inflammation characteristic of Ph-NMPs, predisposing patients to inflammation-driven conditions such as AMD, osteoporosis, rheumatoid arthritis, hypothyroidism, and hepatic disorders [22]. Regarding rheumatoid arthritis, both conditions share the Janus kinase and signal transducer and activator of transcription (JAK-STAT) signaling pathway, driving persistent systemic inflammation and potentially increasing the risk of cardiovascular events. Other conditions, such as ischemic stroke and cardiovascular disease, are linked to thromboembolic events and compromised tissue perfusion. These sequelae stem either from the mechanical effects of elevated cell counts, which increase blood viscosity, or from prothrombotic mediators released by neoplastic cells [23]. Specifically for PV, a 2025 review highlighted an elevated risk of arterial and venous thrombosis, hemorrhage, stroke, myocardial infarction, atrial fibrillation, heart failure, pulmonary hypertension, and leukemic transformation to acute myeloid leukemia [24,25].

During ophthalmologic evaluations, patients did not report subjective low visual acuity (LVA), despite failing to achieve optimal BCVA. This discrepancy is consistent with clinical patterns, as visual impairment rarely manifests as an initial presenting symptom or primary feature of hematologic disease [26]. The fundus evaluation revealed macular drusen, vascular attenuation, and flame-shaped hemorrhages, which corresponded to the clinical diagnoses of AMD (19.4%) and hypertensive retinopathy (9.5%). Notably, flame-shaped retinal hemorrhage was attributed directly to PV when no overlapping systemic or localized ophthalmologic conditions were present. This aligns with the known pathophysiology of PV, wherein hyperviscosity-induced vascular stasis and thrombotic occlusion lead to retinal hemorrhagic changes [24]. In addition, the *JAK2* V617F is known to be more prone to hemorrhagic conditions [27]. Ocular manifestations may be present in about 13.6% of PV cases but are rare as initial symptoms [28]. When analyzing hematological diseases, diagnoses are difficult, especially when ocular manifestations present as initial symptoms or asymptomatically. These changes may be the first manifestations of a systemic disease. Hematological diseases should be ruled out in cases presenting unexplained retinal changes, such as hemorrhages and cotton wool spots [4].

Although the literature presents several ocular manifestations in PV through case reports (such as vascular changes, hemorrhages, transient blindness, papilledema, and occlusions) [29], to the best of our knowledge, there are no major studies promoting a causal link between PV and eye diseases or ocular manifestations [28]. Although a 2022 study reported more prominent posterior segment manifestations in acute leukemia, lymphoma, and multiple myeloma, specifically retinal hemorrhages, posterior segment involvement originating from Ph-NMPs was sparse. In this cohort, NMPs accounted for only 6.57% of patients; among these, 40% presented with ophthalmologic abnormalities, and only 13.34% demonstrated specific retinal findings.

Among posterior segment ocular disorders, AMD predominated, representing 57.1% of identified cases. Accumulated evidence indicates a strong relationship between the development of AMD and chronic systemic inflammation [30]. AMD is a chronic, progressive macular disease characterized by drusen deposition, choroidal neovascularization, or geographic atrophy, whereas Ph-NMPs comprise hematologic disorders driven by distinct cellular, molecular, and cytogenetic aberrations. Notably, both pathologies appear to share convergent inflammatory pathways. A Danish study observed that 234 patients with MPNs were diagnosed with AMD, corresponding to 5.2 cases per 1000 person per year, which can be explained by the presence of common inflammatory mechanisms such as the JAK-STAT signaling modulation [22].

It remains uncertain whether systemic clinical manifestations operate independently of retinal changes or if a larger cohort of eyes with Ph-NMPs must be evaluated to elucidate a potential association. Although published literature associates Ph-NMPs with an increased susceptibility to thrombotic and hemorrhagic events [11], substantial retinal complications were not observed in this cohort. Furthermore, patients presented no subjective complaints of LVA, with the notable exception of a higher prevalence of AMD. Few patients exhibited posterior segment involvement, suggesting that retinal changes are uncommon in individuals with Ph-NMPs, although these patients may still develop concurrent AMD.

Several methodological limitations warrant consideration, including the modest cohort size, data heterogeneity, and limited literature bridging ophthalmic imaging features with systemic outcomes. Owing to the low prevalence of these conditions, the sample size of evaluated eyes is small, underscoring the need for larger multi-center studies to advance future research. Ophthalmological complications in patients with hematological diseases are usually critical and, as in other medical specialties, adequate control of ophthalmological outcomes may indicate improvement in oncological care and can be a barometer for evaluating activities in relation to treatment, prognosis, and quality of care. The evolution of knowledge and the development of new practices form a link between ophthalmology and hematology services, with early identification, immediate risk management, and problem solving. Knowledge and dissemination by multidisciplinary teams, patients, and family members is essential for reducing morbidity, ensuring comprehensive and humane care, although further studies are needed.

## Conclusion

The ophthalmic profile of Brazilian patients harboring Ph-NMPs (typically White, approximately 60 years of age, without sex bias, and well-controlled following early diagnosis) demonstrates a low incidence of subjective LVA and retinal hemorrhagic events despite carriage of the *JAK2* V617F mutation. Nevertheless, these individuals remain susceptible to comorbid AMD alongside other systemic inflammatory conditions.

## Author contributions

Significant contribution to conception and design: RPP, DLCI

Data Acquisition: RPP, MSB, RST, LLN, HLS, JMRSR

Data Analysis and Interpretation: RPP, DLCI

Manuscript Drafting: RPP, DLCI

Significant intellectual content revision of the manuscript: RPP, DLCI, MPA

Have given final approval of the submitted manuscript (mandatory participation for all authors): RPP, DLCI, MSB, RST, LLN, HLS, JMRSR, MPA.

Statistical analysis: RPP

Supervision of administrative, technical, or material support: RPP

Research group leadership: RPP

## Ethics committee of the federal university of Goiás

(CAAE: 79725624.1.0000.5083; Report Number: 6.926.797 of July 2, 2024)

### Data availability

The data that support the findings of this study are available from the corresponding author upon reasonable request.

## Conflicts of interest

All authors declare no conflict of interest.
